# The different clinical characteristics of corona virus disease cases between children and their families in China – the character of children with COVID-19

**DOI:** 10.1080/22221751.2020.1744483

**Published:** 2020-03-25

**Authors:** Liang Su, Xiang Ma, Huafeng Yu, Zhaohua Zhang, Pengfei Bian, Yuling Han, Jing Sun, Yanqin Liu, Chun Yang, Jin Geng, Zhongfa Zhang, Zhongtao Gai

**Affiliations:** a Department of infectious diseases, Jinan Infectious diseases Hospital of Shandong University, Jinan, People’s Republic of China; b Department of Respiratory, Qilu Children’s Hospital of Shandong University, Jinan, People’s Republic of China; c Jinan Institute of Pediatrics, Qilu Children’s Hospital of Shandong University, Jinan, People’s Republic of China

**Keywords:** Coronavirus, severe acute respiratory syndrome coronavirus 2 (SARS-CoV-2), clinical characteristics, corona virus diseases-19 (COVID-19), children

## Abstract

This study aims to analyze the different clinical characteristics between children and their families infected with severe acute respiratory syndrome coronavirus 2. Clinical data from nine children and their 14 families were collected, including general status, clinical, laboratory test, and imaging characteristics. All the children were detected positive result after their families onset. Three children had fever (22.2%) or cough (11.2%) symptoms and six (66.7%) children had no symptom. Among the 14 adult patients, the major symptoms included fever (57.1%), cough (35.7%), chest tightness/pain (21.4%), fatigue (21.4%) and sore throat (7.1%). Nearly 70% of the patients had normal (71.4%) or decreased (28.6%) white blood cell counts, and 50% (7/14) had lymphocytopenia. There were 10 adults (71.4%) showed abnormal imaging. The main manifestations were pulmonary consolidation (70%), nodular shadow (50%), and ground glass opacity (50%). Five discharged children were admitted again because their stool showed positive result in SARS-CoV-2 PCR. COVID-19 in children is mainly caused by family transmission, and their symptoms are mild and prognosis is better than adult. However, their PCR result in stool showed longer time than their families. Because of the mild or asymptomatic clinical process, it is difficult to recognize early for pediatrician and public health staff.

## Introduction

In late 2019, an outbreak of pneumonia with unknown etiology was found in Wuhan, Hubei province, China. Then the pathogen was isolated soon and named the 2019 novel coronavirus (2019-nCoV) on 12 January 2020 [1] And on 11 February, the International Committee on Taxonomy of Viruses announced that its official classification is severe acute respiratory syndrome coronavirus 2 (SARS-CoV-2). The virus spread very fast in Wuhan. Even more unfortunate, as the Chinese Spring Festival is approaching, aggregation of large numbers of people flow caused it to spread quickly across the country and even spread to more than 100 countries [2]. The current case reports are mainly concentrated in Hubei Province and adults, but cases of children outside Hubei Province are rare. Meanwhile, the clinical characteristics of cases in Hubei Province and other provinces were significantly different. Here, we report the clinical manifestations, laboratory test results, imaging characteristics, and treatment regimen of nine SARS-CoV-2 infected children and their families in Jinan, Shandong province to increase awareness of this disease, especially in children.

## Methods

### General information

A retrospective review was conducted of the clinical, lab tests, and radiologic findings for nine children and their families admitted to the Jinan Infectious Diseases Hospital identified to be nucleic acid-positive for SARS-CoV-2 from 24 January 2020 to 24 February 2020. Sample collection and pathogen identification after admission to the hospital, respiratory tract samples including sputum and nasopharyngeal swabs were collected from the patients, which were tested for influenza, avian influenza, respiratory syncytial virus, adenovirus, parainfluenza virus, *Mycoplasma pneumoniae* and chlamydia, along with routine bacterial, fungal, and pathogenic microorganism tests. Real-time PCR used the SARS-CoV-2 (ORF1ab/N) nucleic acid detection kit (Bio-germ, Shanghai, China) and performed refer to previous literature [3]. All the patients were recorded with basic information and epidemiological histories [4] including (1) History of travel or residence in Wuhan and surrounding areas or other reported cases within 14 days of onset; (2) History of contact with new coronavirus infection (nucleic acid-positive) 14 days before onset; (3) history of contact with patients with fever or respiratory symptoms from Wuhan and surrounding areas, or from communities with case reports within 14 days before onset; (4) Cluster onset, along with disease condition changes.

### Laboratory test

Laboratory test results were compiled, including standard blood counts, blood biochemistry, C-reactive protein (CRP), procalcitonin (PCT), erythrocyte sedimentation rate(ESR), Interleukin-6 (IL-6) and myocardial enzyme spectrum. Additional data collected included medical imaging, treatment regimens, and prognosis (any severe complications, including death), and recover or discharge date (Table 1).

**Table 1.** General information of the nine children infected by SARS-CoV-2 in Jinan.

Case no.	Gender	Age	Admit date	Symptom	Contact history	Physical examination	CT scan/X ray	Changes in nucleic acids	Prognosis	The families with COVID	Other people were infected
1	F	2y 9m	25 Jan	No	Father went to Wuhan on 24 Jan	Negative	Negative	7 Feb NT (−), 8 Feb NT (−), 23 Feb ST (+)	Discharge on 9 Feb, Return on 23 Feb	Father and grandma	Father’s colleague
2	F	3y 7m	26 Jan	Fever (37–37.6°C)	Mather went to Wuhan (11–16 Jan)	Negative	Bronchitis	4 Feb NT (−), 5 Feb NT (+), 7 Feb NT (−), 8 Feb NT (−), 23 Feb ST (+)	Discharge on 9 Feb, Return on 23 Feb	Mother, father and grandma	No
3	F	8y 1m	30 Jan	Fever (38.5°C one time)	Father took train many times (15–22 Jan)	Negative	Negative	7 Feb NT (−), 8 Feb NT (−), 23 Feb ST (+)	Discharge on 9 Feb, Isolate at home	Father	Father’s three colleagues
4	M	3y 7m	01 Feb	No	Father transfer the flight in Wuhan on 14 Jan (stay 1 day)	Negative	Bronchopneumonia	7 Feb NT (+), 17 Feb NT (−), 18 Feb NT (−), 23 Feb ST (+)	Discharge on 19 Feb, Return on 23 Feb	Father and grandma	No
5	F	5y 7m	02 Feb	Mild, dry cough	Mother (contacted a friend came from Hubei, 20–25 Jan)	Negative	Negative	2 Feb NT (+), 8 Feb NT (+), 12 Feb NT (−), 13 Feb NT (−).	Recover	Mather and father	No
6	F	5y 2m	03 Feb	No	Father went to Wuhan (18–21 Jan)	Negative	Negative	3 Feb NT (+), 7 Feb NT (+), 17 Feb NT (−), 18 Feb NT (−)	Recover	Father	No
7	M	11m	06 Feb	No	Parents work in Wuhan and went home on 22 Jan	Negative	Pulmonary consolidation and GGO	7 Feb NT (+), 13 Feb NT (+), 17 Feb NT (−), 18 Feb NT (−), 23 Feb ST (+)	Discharge on 20 Feb, Return on 23 Feb	Grandparents, Parents and brother	No
8	M	11m	06 Feb	No	Parents work in Wuhan and went home on 22 Jan	Negative	Negative	7 Feb NT (+), 13 Feb NT (+), 17 Feb NT (−), 18 Feb NT (−), 23 Feb ST (+)	Discharge on 20 Feb, Return on 23 Feb	Grandparents, Parents and brother	No
9	F	9y	10 Feb	No	Father (negative history)	Negative	bronchitis	9 Feb NT (+), 18 Feb NT (−).	Recover	Father	No

Note: NT, Nasal and throat swabs; SS, Sputum specimen (all the children no sputum); ST, stool; GGO, ground glass opacity.

### Ethics

This study was conducted in accordance with the Declaration of Helsinki. Informed consent was waived because of the retrospective nature of the study and the analysis used anonymous clinical data.

### Data analysis

Continuous data are expressed as medians and ranges, and categorical data are presented as counts and percentages.

## Results

### General information

There were three boys, six girls and their 14 families admitted to Jinan Infectious Disease Hospital of Shandong University were investigated in this study. The youngest of the nine children was a pair of eleven-month-old twins and the oldest is nine years and 9 months old (mean age was 4.5 years, median age 3.5 years, Table 1). There were 16 families were infected by SARS-CoV-2, and 14 adults were enrolled in this study (two patients hospitalized in another hospital). The 14 patients consisted of 8 males and 6 females with a mean age of 42.9 years (median age, 37 years [range, 30–72 years]).

### Clinical manifestations

All nine pediatric patients came from eight families. As shown in Table 1, six children had no information on symptoms available, but have positive results in nucleic acid detection after the positive diagnosis of their families. By contrast, only one child has wild cough and two children have a mild fever (37.4–38.5°C). None of the nine children required intensive care or mechanical ventilation or had any severe complications.

For the 14 adult patients, the main clinical symptoms were fever (8/14, 57.1%), cough (5/14, 35.7%), chest tightness/pain (3/14, 21.4%), fatigue (3/14, 21.4%) and sore throat (1/14, 7.1%). Meanwhile, there were four patients had no clinical symptoms. From the epidemiological data, 7/14(50%) of the adults were infected through household contact, 5 (35.8%) was found to be infected after returning from Wuhan or Hubei in late January 2019 and 2 (14.2%) patients couldn’t find the exact source of infection.

### Laboratory tests and imaging examinations

**Figure 1:**
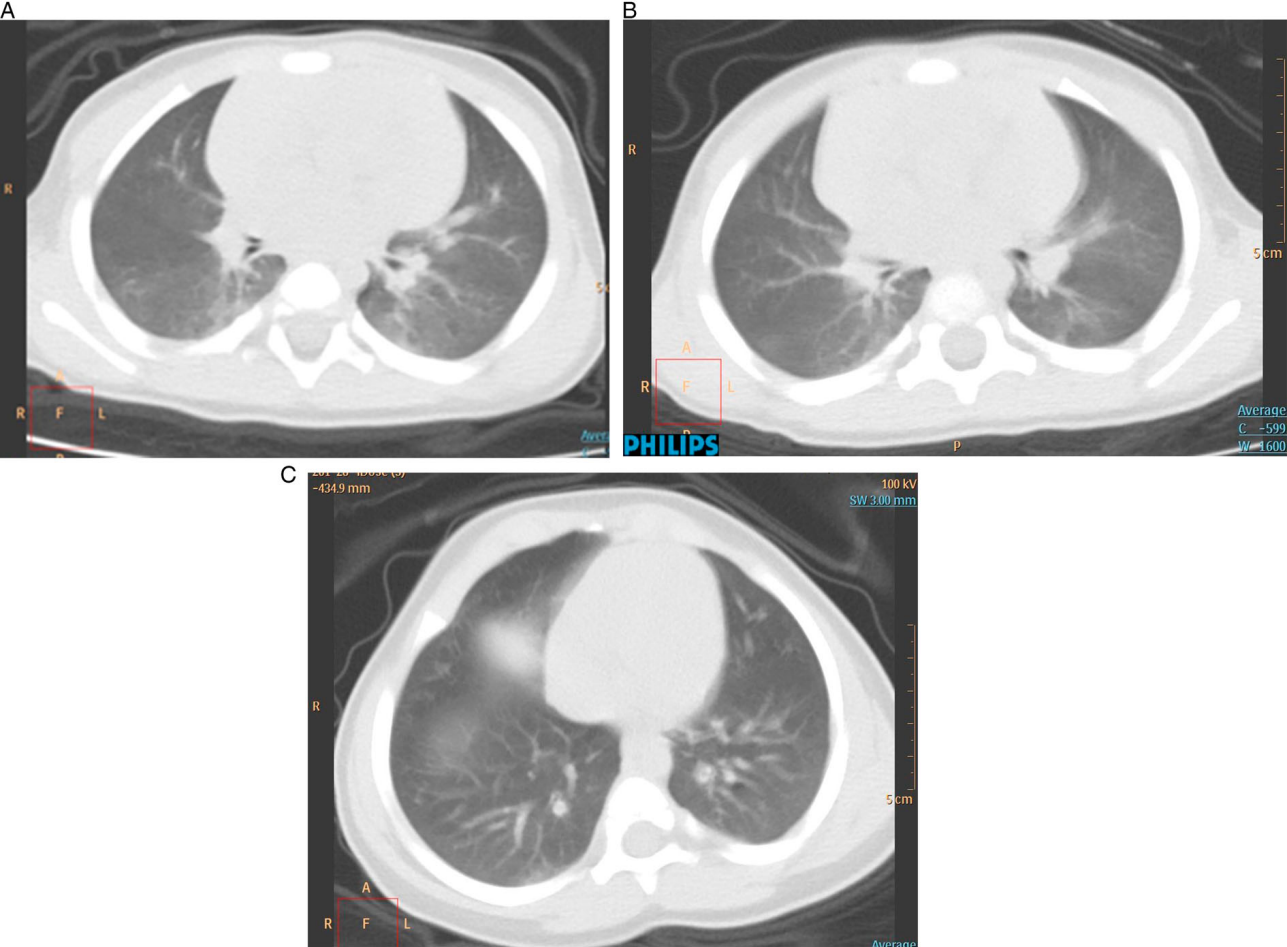
Chest CT images of a case 7 and case 8 (one pair of 11 month old twins) patient upon admission, who had no symptom. (A) Transverse chest CT images showed pulmonary consolidation and ground glass opacity (case 7 on 7 February). (B) Showed marked improvement after 5 days (case 7 on 13 February). (C) Lung CT of case 8 – the case 7’s younger brother on admission day (case 8 on 7 February).

As shown in Table 2, 8/9 (88.9%) children had normal or decreased white blood cell counts, consistent with the main characteristic of viral infection. Six children (66.7%) showed increased CK-MB. ALT, AST and the other index of liver and kidney were all normal. All inflammation indicators, including CRP, PCT, ESR and IL-6 were all within the normal range. Two children (22.2%) showed bronchitis and one (11.1%) showed bronchial pneumonia. One (11.1%) boy (the older of the twins) showed pulmonary consolidation and ground glass opacity on the first day (Figure 1(A)) admitted in the hospital, and disappeared after five days (Figure 1(B)). Five other (55.6%) children showed no abnormal chest radiograph.

**Table 2.** The laboratory results of nine children patients.

Case no.	WBC (10^9^/L)	N (%)	L (%)	PLT (10^9^/L)	Hb (g/L)	CRP (mg/L)	ESR (mm/h)	PCT (ug/L)	IL-6 (pg/ml)	ALT (U/L)	AST (U/L)	CK-MB (U/L)	CD4 (%)	CD8 (%)	NK (%)	IgM (g/L)	IgA (g/L)	IgG (g/L)	C3 (g/L)	C4 (g/L)	Ferritin	D-dimer	Myoglobin (ug/l)	BNP (pg/ml)	SAA
1	8.48	32.1	59.9	361	128	0.12	5	0.04	1.5	13	35	34	31.9	34.6	16.5	1.0	0.6	7	0.8	0.2	45.5	0.3	9	133	3
2	7.55	20.4	73.6	193	120	0.35	2	0.08	1.5	14	33	28	28.3	27.1	27.3	1.2	0.8	7.3	0.8	0.3	33.6	0.3	11	24	4
3	3.78	38.7	43.9	293	119	0.19	7	0.05	1.5	15	28	23	33.2	35.6	–	1.1	1.6	12.9	1.0	0.2	85.6	0.4	9	29	3
4	4.14	23.6	69.3	65	129	0.18	6	0.04	1.5	14	28	30	46.6	20.2	8.5	1.1	0.8	6. 9	1.0	0.2	–	0.2	9	116	5
5	3.69	33.8	53.3	169	106	0.12	5	0.03	1.5	15	23	22	45.7	33.4	14,8	0.9	0.6	8.1	0.1	1.0	48.1	0.1	9	87	3
6	9.33	27.8	66.7	278	133	0.24	2	0.08	1.5	13	36	76	36.9	22.6	16.7	1.6	1.7	16.3	1.0	0.2	50.7	0.2	9	39	6
7	11.17	17.2	77.4	305	117	0.19	2	0.04	1.5	22	39	43	–	–	–	1.3	0.3	7.1	0.7	0.1	62.6	0.3	9	31	3
8	9.42	24.5	68.6	358	116	0.19	2	0.04	1.5	22	42	52	–	–	–	0.6	0.2	7.0	0.7	0.1	59.7	0.3	9	2	3
9	5.45	41.2	53.0	235	140	0.26	3	0.02	1.5	9	24	27	33.1	29.3	16.5	1.3	1. 6	8.9	1.0	0.2	45.6	0.3	18	23	5

Note: WBC, white blood cell; N, Neutrophil; L, Lymphocyte; PLT, plate; Hb, Hemoglobin; ESR, erythrocyte sedimentation; PCT, Procalcitonin; ALT, alanine aminotransferase; AST, aspartate aminotransferase; CK-MB, creatine kinase MB; IgM, Immunoglobulin M; IgG, Immunoglobulin G; Ig, Immunoglobulin A; C3, complement3; C4, complement4; BNP, N-terminal brain natriuretic peptide precursor; SAA, Serum amyloid A.

**Figure 2.**
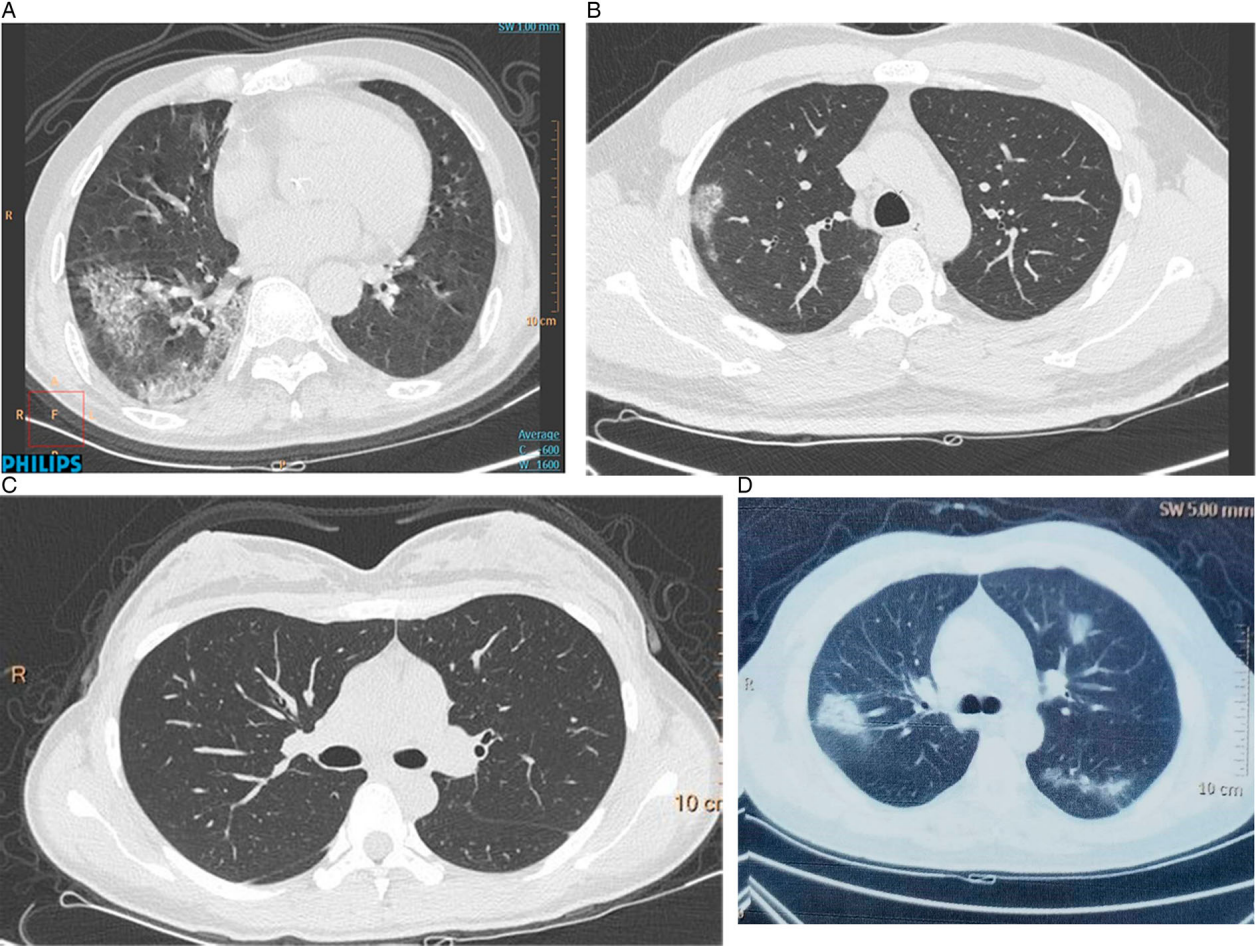
Lung computed tomography (CT) images of case 7 and case 8’s families. (A) The CT of their grandpa, who was 72-year-old and admitted for symptoms of high fever for 3 days. (B) CT scan of case their father, a 38-year-old patient admitted for symptoms of high fever, shortness of breath and fatigue for 10 days on his admission day. (C) Lung CT image of their mother, a 32-year-old female patient admitted for positive result of PCR (C). (D) Chest CT of the twins’ grandma, 65 years old and had fever, dry cough and chest pain for 6 days before admission.

All the adult patients had normal (10/14, 71.4%) or decreased (4/14, 28.6%) white blood cell counts and 10 (71.4%) have lymphopenia. There were 4 (28.6%) patients had increased CRP, PCT, Serum amyloid A (SAA), D-dimer and IL-6, meanwhile, their CT-scan showed larger lung consolidation. Compared to children, there were only two (14.3%) patients showed increased CK-MB. Ferritin in the adult patients were higher than the children but most of them were normal (11/14, 78.6%). The imaging of adult chest was mix and the most common characters of imaging were pulmonary consolidation (50%), nodular shadow (42.9%), and ground glass opacity (GGO, 35.7%) (Figure 2). Four (28.6%) adults showed normal chest imaging.

### Treatment regimen and prognosis

At present, there are no drugs available that can target SARS-CoV-2. Therefore, treatment was focused on symptomatic and respiratory support. All the children inhaled interferon and one of the twins was prescribed ribavirin (10–15 mg/kg.d) in addition. Ten (71.4%) adults with pneumonia were treated Lopinaviritonavir (200/50 mg, 2 tablets, bid), interferon and Chinese medicine. The patients with higher infection index (such as CRP, PCT, ESR, SAA, IL-6) were prescript antibiotics for 5–7 days in addition. All the nine children and 14 adult patients recovered in 2–3 weeks and were discharged after two negative nucleic acid tests. Unfortunately, our follow up found that there were five discharged children were admitted again before we submit this article because their stool showed positive result in SARS-CoV-2 PCR. Meanwhile, all their families were negative in all the specimen.

## Discussion

Coronaviruses are a large family of viruses that are known to cause illness ranging from the common cold to more severe diseases. Aa an enveloped RNA virus, Cov is ubiquitous in humans, other mammals, and birds, which can cause respiratory, digestive, liver and nervous system disorders [5,6]. To date, six CoVs have been known to cause human infection [7]. Among them, two zoonotic viruses, SARS-CoV and MERS-CoV, were responsible for serious outbreaks: in China in 2002–2003 [8, 9] and in the Middle East in 2012 [10], respectively. A novel coronavirus was identified in late 2019 in Wuhan, China. This is a new coronavirus that has not been previously identified in humans. On 11 February, The International Committee on Taxonomy of Viruses (ICTV) announced that the official classification of the new coronavirus (2019-nCoV) is called severe acute respiratory syndrome coronavirus 2 (SARS-CoV-2). The World Health Organization (WHO) announced on the same day that the official name of the disease caused by the virus is Corona Virus Disease-19 (COVID-19).

Of particular concern, our observations found that all the children were diagnosed after their families, which indicated that they were infected by the household contact. However, after an epidemiological investigation, we found that six adults (42.9%) had a definite or suspicious contact history and six families (42.9%) contacted them were infected, while the other two patients (14.3%) denied any epidemiological history. Among them, the father of case 9 did not contact anyone who came back from Wuhan or Hubei, but also denied contact with any person with respiratory symptoms. At the same time, through official investigations, they did not find that someone was diagnosed with SARS-CoV-2 infection on the vehicle he was travelling on, prompting the virus to spread. In addition, from the official information, more and more patients can’t find the clue of infection and more and more cluster outbreak showed that no contact, no close communication and even never go out the door. So, we think that these phenomena maybe suggest that: (1) the virus spreads very strongly and the transmission of the virus may not be limited to contact, droplets and airborne transmission, and aerosol transmission may also exist, which was similar to SARS [11]. (2) the virus may be carried asymptomatically after infecting the human body but can infect other people.

In China, the SARS outbreak of 2003 is still impressive, because the 2002–2003 SARS outbreak infected 8422 individuals leading to 916 deaths in eight affected areas [12]. During the SARS outbreak, there were less children patients and the symptoms are significantly milder in children than in adults [13–16]. Similarly, the official data to date suggest that children infected with the SARS-CoV-2 are relatively rare too [17], and their overall symptoms are significantly mild. The main reasons for this phenomenon may be: (1) the range of activities for children is relatively small, they are mainly infected by their adult families. And, as an RNA virus, the SARS-CoV-2 virus maybe also is prone to mistakes in replication, mutating, and surviving without recognition by the immune system, but can also cause a decline in virulence. So, children are infected with second or third generation or even fourth generation virus and they get milder symptoms; (2) it may be because of differences in the immune responses of children compared to adults. One hypothesis is that the innate immune response, that is the early response that is aimed broadly at groups of pathogens, tends to be more active in children. The innate immune system is the first line of defense against pathogens. Cells in that system respond immediately to foreign invaders. The adaptive immune system, by contrast, learns to recognize specific pathogens, but takes longer to join the battle. If the innate immune response is stronger in children exposed to SARS-CoV-2, they may fight off infection more readily than adults, suffering only mild symptoms. Other coronaviruses, including SARS and MERS, also show this pattern [18]. (3) The number or function of ACE2 receptors in children is not as good as in adults. Recently, one studies had investigated the role of the ACE2 receptor and found that the SARS-CoV-2 uses the SARS-coronavirus receptor ACE2 and the cellular protease TMPRSS2 for entry into target cells [19]. As we know, the distribution of ACE2 receptors in different organs and populations is different. Therefore, it may be that different receptor levels or functions in children and adults lead to different severity of illness. (4) Other reasons: such as children have fewer basic diseases, children smoke less, and children have strong self-healing capabilities and so on.

CK-MB is an indicator of myocardial injury. In the present study, we found six children and two adults had high CK-MB, which means that SARS-CoV-2 can cause heart injury. It is reported that the main mechanisms of SARS-CoV-2-induced myocardial injury may be the direct injury of virus, the inflammatory storm and the distribution of ACE2 receptor [20].

As human lifestyles change, more and more viruses are spreading across species. Current research confirms that SARS-CoV-2 are transmitted from animals to humans. Like other viruses, the relationship between SARS-CoVs and humans has the following possibilities: (1) the virus disappears for some unknown reasons, such as SARS-CoV. (2) Viruses coexist with humans and have seasonal onsets, such as flu influenza viruses. The first is the best outcome of the current situation, but the second possibility is very large. If, as we analyzed above, many people, especially children with mild or no clinical symptoms carry the virus but do not develop the disease, however, the virus spread very strongly, it may lead to the silent spread of the disease and leading to major losses. Therefore, the Chinese government will face greater risks after school starts and work resumes. And, clinicians, especially pediatricians, need to be vigilant to prevent widespread spread of the disease. Children who have infected family members should be monitored or evaluated and family clustering should be reported to ensure a timely diagnosis.

In addition, just before we submit, we found that five of six discharged children returned to the hospital because of positive PCR in their stool, however, their families were all negative. One girl (case 3) didn’t return to the hospital but isolated in home because she had mild mental symptoms after discharge. Although positive results cannot confirm there were live virus in the stool or not. However, for insurance of public health, they were admitted to the hospital again to get clinical observation. Interestingly, their onset was later than their families, but the period of positive PCR was longer than adults. We should pay more attention to this phenomenon and study the possible mechanism.

Several important limitations of this study should be noted. First, the size was small. Second, the retrospective study included only of children who were hospitalized in one hospital. But as one of the rare reports in children out Hubei province, it’s helpful to improve the ability to recognize patients with mild illness. Further studies with large multi-center samples are needed.

In conclusion, by analyzing 23 confirmed cases of COVID-2019 in Jinan, Shandong province, this study’s findings indicate that new control measures should include rapid medical assessment and removal of the case from the home, as well as increased awareness of the importance of protective measures after symptom onset. Public health measures such as home isolation should be aimed at minimizing such risk factors when addressing household transmission of serious infections spread through droplet transmission.

## References

[1] Novel coronavirus (2019-nCoV). Geneva: World Health Organization; 2019. Available from: https://www.who.int/emergencies/diseases/novel-coronavirus.

[2] WHO. Coronavirus disease (COVID-2019) situation reports. Available from: https://www.who.int/docs/default-source/coronaviruse/situation-reports/20200310-sitrep-50-covid-19.pdf?sfvrsn=55e904fb_2.

[3] Kui L, Fang YY, Deng Y, et al. Clinical characteristics of novel coronavirus cases in tertiary hospitals in Hubei province. Chin Med J (Engl). 2020. DOI:10.1097/CM9.0000000000000744. PMC7147277 32044814

[4] National Health Commission of People’s Republic of China. Diagnosis and treatment of new coronavirus pneumonitis. 5th ed. (Chinese). Available from: http://117.128.6.32/cache/www.nhc.gov.cn/jkj/s3577/202002/a5d6f7b8c48c451c87dba14889b30147/files/3514cb996ae24e2faf65953b4ecd0df4.pdf?ich_args2=464-11172813036679_88eae94af1a195e2d387e01ae83b27b9_10001002_9c896c2fdec2f9d99f38518939a83798_c8745eab2a416ddd81cb9150f1f76daf.

[5] Weiss SR, Leibowitz JL. Coronavirus pathogenesis. Adv Virus Res. 2011;81:85–164. DOI:10.1016/b978-0-12-385885-6.00009-2. 22094080 PMC7149603

[6] Zhou P, Fan H, Lan T, et al. Fatal swine acute diarrhoea syndrome caused by an HKU2-related coronavirus of bat origin. Nature. 2018;556:255–258. DOI:10.1038/s41586-018-0010-9. 29618817 PMC7094983

[7] Su S, Wong G, Shi W, et al. Epidemiology, genetic recombination, and pathogenesis of coronaviruses. Trends Microbiol. 2016;24:490–502. DOI:10.1016/j.tim.2016.03.003. 27012512 PMC7125511

[8] Normile D. Infectious diseases. Battling SARS on the frontlines. Science. 2003;300:714–715. DOI:10.1126/science.300.5620.714. 12730562

[9] Zhong NS, Zheng BJ, Li YM, et al. Epidemiology and cause of severe acute respiratory syndrome (SARS) in Guangdong, People’s Republic of China, in February, 2003. Lancet. 2003;362:1353–1358. DOI:10.1016/s0140-6736(03)14630-2. 14585636 PMC7112415

[10] Zaki AM, Van Boheemen S, Bestebroer TM, et al. Isolation of a novel coronavirus from a man with pneumonia in Saudi Arabia. N Engl J Med. 2012;367:1814–1820. DOI:10.1056/NEJMoa1211721. 23075143

[11] Booth CM, Matukas LM, Tomlinson GA, et al. Clinical features and short-term outcomes of 144 patients with SARS in the greater Toronto area. JAMA. 2003;289:2801–2809. doi: 10.1001/jama.289.21.JOC30885 12734147

[12] Summary of probable SARS cases with onset of illness from 1 November 2002 to 7 August 2003. Available from: http://www.who.int/csr/sars/country/country2003_08_15.pdf.

[13] Ng PC, Leung CW, Chiu WK, et al. SARS in newborns and children. Biol Neonate. 2004;85:293–298. DOI:10.1159/000078174. 15218286

[14] Chiu WK, Cheung PCH, Ng KL, et al. Severe acute respiratory syndrome in children: experience in a regional hospital in Hong Kong. Pediatr Crit Care Med; 2003;4:279–283. doi: 10.1097/01.PCC.0000077079.42302.81 12831407

[15] Hon KL, Leung CW, Cheng WT. Clinical presentations and outcome of severe acute respiratory syndrome in children. Lancet. 2003;361:1701–1703. doi: 10.1016/S0140-6736(03)13364-8 12767737 PMC7112484

[16] Spicuzza L, Spicuzza A, La Rosa M, et al. New and emerging infectious diseases. Allergy Asthma Proc. 2007;28:28–34. DOI:10.2500/aap.2007.28.2870. 17390754

[17] The Novel Coronavirus Pneumonia Emergency Response Epidemiology Team. The epidemiological characteristics of an outbreak of 2019 novel coronavirus diseases (COVID-19) in China. Chin J Epidemiol. 2020;41(2):145–151. DOI:10.3760/cma.j.issn.0254-6450.2020.02.003. PMC8392929 34594836

[18] Available from: https://www.livescience.com/why-kids-missing-coronavirus-cases.html.

[19] Hoffmann M, Kleine-Weber H, Krüger N, et al. The novel coronavirus 2019 (2019-nCoV) uses the SARS-coronavirus receptor ACE2 and the cellular protease TMPRSS2 for entry into target cells. Available from: https://www.biorxiv.org/content/10.1101/2020.01.31.929042v1.

[20] Wu CM, Hu XL, Song JX, et al. Heart injury signs are associated with higher and earlier mortality in coronavirus disease 2019 (COVID-19). medRxiv. 2020. DOI:10.1101/2020.02.26.20028589.

